# Comparison of Effect of Two-Hour Exposure to Forest and Urban Environments on Cytokine, Anti-Oxidant, and Stress Levels in Young Adults

**DOI:** 10.3390/ijerph13070625

**Published:** 2016-06-23

**Authors:** Su Geun Im, Han Choi, Yo-Han Jeon, Min-Kyu Song, Won Kim, Jong-Min Woo

**Affiliations:** 1 Department of Psychiatry, Seoul Paik Hospital, College of Medicine Inje University, Seoul 04551, Korea; leo0101j@gmail.com (S.G.I.); smk-style@hanmail.net (M.-K.S.); phrenie@naver.com (W.K.); 2 Graduate School of Art Therapy, Cha University, Sungnam 11160, Korea; hanchoi.stress@gmail.com; 3 Department of Child Psychology and Education, Sungkyunkwan University, Seoul 110-745, Korea; creativitylex@gmail.com; 4 Korea Employee Assistance Professionals Association, Seoul 04551, Korea

**Keywords:** forest, cytokines, glutathione, interleukin, antioxidant, crossover design

## Abstract

The purpose of this study was to investigate the effect of two-hour exposure to a forest environment on cytokine, anti-oxidant and stress levels among university students and to compare the results to those measured in urban environments. Forty-one subjects were recruited. For our crossover design, subjects were divided into two groups based on similar demographic characteristics. Group A remained in the urban environment and was asked to perform regular breathing for 2 h. Blood samples were collected and the serum levels of cytokines including interleukin-6 (IL-6), IL-8, tumor necrosis factor-α (TNF-α), and glutathione peroxidase (GPx) were examined. Subjects were moved to a small town in a rural area for an equal amount of time to exclude carryover effects, and then remained for another 2 h in a forest environment. The second set of blood samples was collected to assess the effect of exposure to the forest environment. Using the same method, Group B was first exposed to the forest environment, followed by exposure to the urban environment. Blood samples collected after the subjects were exposed to the forest environment showed significantly lower levels of IL-8 and TNF-α compared to those in samples collected after urban environment exposure (10.76 vs. 9.21, *t* = 4.559, *p* < 0.001, and 0.97 vs. 0.87, *t* = 4.130, *p* < 0.001). The GPx concentration increased significantly after exposure to the forest environment (LnGPx = 5.09 vs. LnGPx = 5.21, *t* = −2.039, *p* < 0.05).

## 1. Introduction

People who have suffered from extremely high levels of stress, such as those due to the loss of their loved ones, are generally in a state of inferior health. According to psycho-immunology, a pathologic lesion located in the hypothalamus, the hippocampus, or the pituitary gland can cause abnormalities in the immune system [1,2]. Lymphocytes secrete adrenocorticotropic hormone and endorphins, which significantly affect the central nervous system [3]. A number of studies have examined the effect of stress and mental diseases on the immune system [4,5]. Since the levels of immune cells showing immunological changes can be easily assessed in peripheral blood samples, investigating the molecular biological changes resulting from stress is an effective approach for understanding the relationship between stress and the immune system. Additionally, neurological changes resulting from the interaction between serotonin and cytokines of the immune system may affect the pathophysiology of depression [6]. Psychological stress results in decreased levels of tryptophan, a precursor of serotonin, and may cause mood swings. With persistent psychological stress, the level of pro-inflammatory cytokines begins to increase. In response, the body attempts to balance the immune system with negative feedback mechanisms by increasing the secretion of anti-inflammatory cytokines. However, once the enzyme indoleamine 2,3-dioxygenase is activated and the metabolism of tryptophan to kynurenine is accelerated, serotonin levels in the brain decrease and the subject becomes susceptible to depression [4].

A number of studies have suggested that psychiatric disorders are related to inflammatory diseases [7,8,9]. Increased oxidative stress causes inflammation, and more importantly, stimulates microglia in the brain. The inflammatory response resulting from microglial activation can progress to brain pathology and influence treatment responses [4]. Microglial activation can be divided into two distinct actions: classical M1 and alternative M2 activation [10]. Microglial cells can synthesize pro-inflammatory molecules such as interleukin-1β (IL-1β), tumor necrosis factor-α (TNF-α), and IL-6 and IL-8, which ultimately resolve infections and facilitate tissue recovery [11,12]. M2 activation can be induced by cytokines such as IL-4, IL-13, and IL-25 [10,13], which are associated with the release of anti-inflammatory cytokines such as IL-10, insulin-growth factor-1, transforming growth factor-β, and neurotrophic factors. M2 activation facilitates healing and prohibits neuronal injury [14,15]. IL-8 mainly acts as a neutrophil and lymphocyte chemoattractant and activation factor. TNF-α enhances T and B cell proliferation and differentiation, and increases Natural killer (NK) cell activity [16]. Cytokines are small glycoproteins that mediate signal communications among various immune and neuronal cells. Th1 cells have been associated with cellular immunity against intracellular bacteria and viruses, and even autoimmune diseases such as multiple sclerosis and rheumatoid arthritis. Moreover, Th2 cells manage hormonal immunity against extracellular parasites and allergic reactions [17,18].

The family of glutathione peroxidases (GPx) contains four distinct mammalian selenoproteins [19]. Cytosolic or classical glutathione peroxidase is universally distributed and functions to block oxidative attack [20,21]. The gastrointestinal isoenzyme of GPx is exclusively located in the gastrointestinal tract. It may act as a barrier against hydroperoxides originating from the diet or metabolism of ingested xenobiotics [22]. Plasma GPx is similar to cytosolic GPx and is produced during selenium deficiency [23]. Plasma GPx resides in extracellular compartments and is expressed in various tissues such as the ciliary body, the kidney, and maternal/fetal interfaces in contact with body fluids. Phospholipid hydroperoxide glutathione peroxidase is a ubiquitous antioxidant enzyme that protects membrane lipids [24].

One study revealed that forest environments have beneficial physiological effects on patients with allergies or respiratory diseases [25]. Factors in forests that are regarded to have beneficial physiological effects include plant aroma, temperature, humidity, light intensity, wind, and oxygen concentration [26]. Compared to forest environments, urban environments contain higher levels of air pollutants such as dust, nitrogen oxide, and carbon oxide [27]. Air pollution substances can also affect blood cells and the immune system, including white blood cells, red blood cells, monocytes, and lymphocytes [28]. Phytoncide, a terpene substance containing essential oils from plants, is a biogenic volatile organic compound that is abundant in forest environments [29]. A previous study has suggested that refined essential oils or D-limonene from plants can improve immune function [30]. Even over a two-night trip to a broad-leaf evergreen forest, oxidative stress and pro-inflammatory levels were lower than in the control group who remained in the city during the same period [31]. However, no studies have examined the effect of a brief 2 h exposure to a forest environment on the immune system. In the present study, we investigated the positive effects of a 2 h exposure to a forest environment on cytokine and anti-oxidant levels in university students experiencing moderate levels of stress.

## 2. Materials and Methods

### 2.1. Experimental Sites

This study was conducted in the pine tree forest “Jat-Hyanggi Green Forest” (Gyeonggi Province, Korea). “Jat-Hyanggi” means “the scent from Pinus koraiensis” which is abundant in that area and make up a typical coniferous forest in northern area of Korean peninsula, and Gyeonggi Province is easy to access from Seoul. One previous study surveyed 12 forest sites in Gyeonggi Province, and “Jat-Hyanggi Green Forest” has the highest level of phytoncide, annual average 1.251 µg/m^3^, compared to other sites. The period from June to August showed the highest level of phytoncide [32]. To maximize the effect of exposure to forest environment, we conducted the experiment in June. We chose Seoul Paik hospital for urban environment exposure and the rural area of Hwado-eup (Gyeonggi Province, Korea) for wash-out period.

### 2.2. Subjects

From 21 March 2015 to 31 May 2015, an initial survey was conducted for 138 young adults aged between 18 and 35 years who attended two universities in Seoul. Forty-one subjects who met the initial survey selection criteria and voluntarily agreed to participate in the study were enrolled. Subjects were recruited through advertisement and initially assessed the following descriptions: (1) The subject experiences a change in mood in response to a change in the environment; (2) The subject is capable of living a normal life with the help of others; (3) The subject is adequately interested in his or her surroundings; and (4) The subject can recognize changes in his state of health and requires support from others when his or her work load is heavy. Subjects who met the following criteria were included: (1) The subject experiences a higher than average level of stress; (2) The subject is an undergraduate or graduate student aged 18–35 years; and (3) The subject’s residential address is in a metropolitan area. Exclusion criteria included: (1) The subject has a diagnosed psychiatric disease; (2) The subject has plant- or pollen-related allergic rhinitis or chronic allergic rhinitis; and (3) The subject has bronchial asthma. None of the subjects reported any medical history of physiological or psychiatric disorders.

To conduct this crossover design experiment, the recruited experimental group showing the same demographic characteristics was divided into two groups. A simple and unrestricted randomization method was used to maintain a 1:1 assignment into each group. Using a random number generator, numbers ranging from 1 to 41 were apportioned by utilizing random place cards. Analysis of the demographics of the participants (Table 1) revealed no significant differences in any of the variables, with the exception of alcohol consumption. Biserial correlation analysis was performed in order to test alcohol intake may have influenced the result. The result revealed that there was no significant correlation between alcohol intake and each variables. Thus, groups A and B were determined as the cohorts.

The study was approved by the Seoul Paik Hospital’s Institutional Review Board (IIT-2015-207). The study was fully explained to all subjects in both spoken and written form, specifically focusing on its purpose, the precise procedures that would be used, and any possible adverse events. Informed consent was obtained from all subjects.

### 2.3. Procedure

Subjects who agreed to participate in the survey were given an identification number, and the numbers were recorded on the screening survey printouts. These numbers were used to identify subjects who completed the survey without the possibility of acquiring personal information about the subjects. After obtaining permission from the IRB (IIT-2015-207), the subjects who met our inclusion criteria and voluntarily expressed interest in the study were asked to participate.

The experiment was conducted on 22 June 2015. On the day before the experiment, all subjects were fully informed of the experimental purpose and procedure. The experimental schedule is shown in Table 2. This study applied a cross-over design, which is a method used to apply both treatments to the same subject. This method is advantageous because research can be conducted using only the study group without the need for a control group, thus increasing efficiency [33]. In this study, the experimental group was exposed to an urban environment followed by exposure to a forest environment for 2 h to observe psychological and biological changes. In addition, in order to reduce carryover effects, a 2 h wash-out period was included between the two treatments.

In group A, all subjects gathered in the same location for exposure to the urban environment and remained for 2 h. After exposure, we collected blood samples from all the subjects to examine the serum levels of cytokines, including IL-6, IL-8, TNF-α, and glutathione peroxidase (GPx). Subjects were sent to a small town in the rural area for an equal amount of time to reduce carryover effects, followed by exposure to a forest environment for 2 h. Next, we collected the second set of blood samples to assess the effect of exposure to a forest environment. Group B was initially exposed to the forest environment followed by exposure to the city environment by using the same methods.

Control variables for the subjects were as follows: (1) Restriction of alcohol intake for 12 h prior to experiment participation; (2) Restriction of food intake for 1 h prior to experiment participation; (3) Restriction of smoking and exhilarating drinks throughout the duration of experiment participation; (4) Forest therapy program which included therapeutic factors was not implemented; (5) Restriction of excessive movement or individual activity and behavior outside the test area; (6) Restriction of excessive use of mobile devices or learning activities; (7) Provision of same meals and drinks throughout the duration of the experiment participation period; (8) Measurement of pre- and post-analysis for all subjects at the same time.

### 2.4. Psychological Indices

In this study, the stress response inventory-modified form (SRI-MF) was utilized as described in Koh et al. [34], and the scale was modified based on the workplace [35]. SRI-MF measures experiences with respect to stress, particularly the mental health and physical symptoms related to stress over a period of one week. The survey is composed of three subcategories, including somatic symptoms, depression, and anger, with a total of 26 questions. Each question consists of a five-point scale. A measure of SRI-MF with score ranging 0–31 = low level of stress response, 32–49 = moderate level of stress response, above 50 = high level of stress response.

### 2.5. Physiological Indices

The serum levels of IL-6, IL-8, TNF-α (cytokine), were assessed using an enzyme-linked immunosorbent assay with the BD OptEIA kit (BD Biosciences, Franklin Lakes, NJ, USA). Cayman’s GPx assay, that we used, measures GPx activity indirectly by a coupled reaction with glutathione reductase. Oxidized glutathione (GSSG), produced upon reduction of hydroperoxide by GPx, is recycled to its reduced state by GR and NADPH. The Cayman GPx Assay Kit can be used to measure all of glutathione-dependent peroxidases in plasma, erythrocyte lysates, tissue homogenates, and cell lysates. We collected blood samples from all of subjects and aimed to measure GPx in plasma and erythrocyte lysate. Using a tube containing anti-coagulant, 5 mL of whole blood was collected.

### 2.6. Statistical Analyses

The results are expressed as the mean ± SD. Final data analysis was performed using SPSS version 21.0 (IBM Corporation, Armonk, NY, USA). First, to compare the demographic characteristics between groups A and B, the chi-squared test was conducted. Paired *t*-tests were conducted to analyze the differences between exposures to urban and forest environments. A *p*-value of less than 0.05 was considered statistically significant.

## 3. Results

### 3.1. Effect of Exposure to Forest Environment on Stress Levels

**Figure 1:**
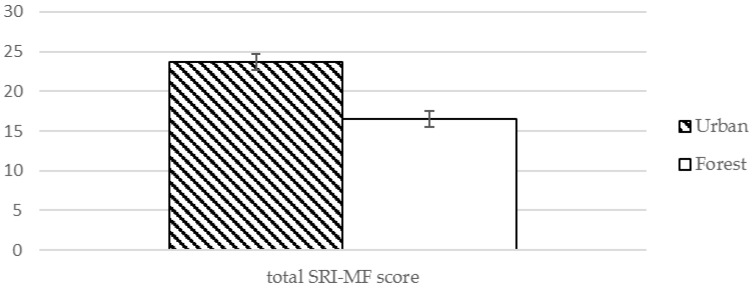
Effect of exposure to forest environment on total SRI-MF score.

The level of somatic symptoms and depressive symptoms were decreased significantly after exposure to the forest environment, and the total score for the stress response was also significantly decreased (Table 3, Figure 1). Anger symptoms were reduced after visiting the forest environment, but the difference was not statistically significant (Table 3).

### 3.2. Effect of Exposure to Forest Environment on Serum GPx and Pro-Inflammatory Cytokine Levels

As shown in Table 4, GPx levels were increased after exposure to the forest environment compared to those after exposure to the urban environment (LnGPx = 5.09 vs. LnGPx = 5.21, *t* = −2.039, *p* < 0.05), which is suggestive of a significant increase of antioxidant level.

**Figure 2:**
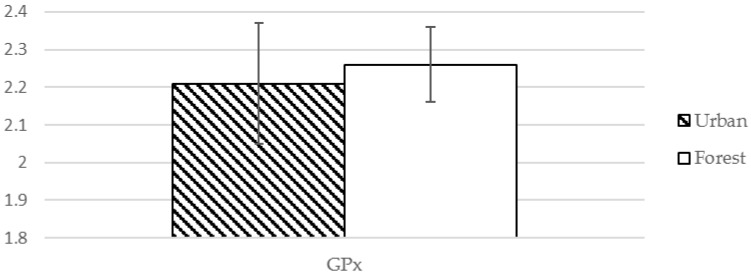
Effect of exposure to forest environment on GPx.

**Figure 3:**
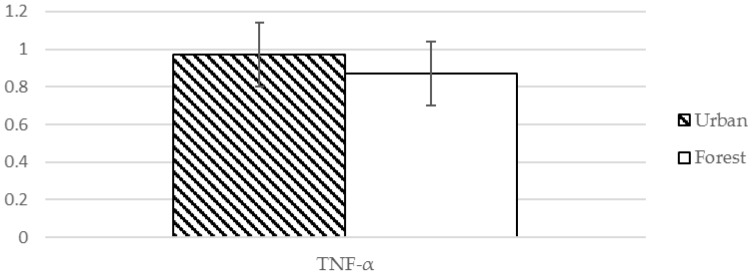
Effect of exposure to forest environment on TNF-α.

Serum IL-8 and TNF-α levels were significantly decreased after exposure to the forest environment compared to those after exposure to the urban environment (10.76 vs. 9.21, *t* = 4.559, *p* < 0.001, and 0.97 vs. 0.87, *t* = 4.130, *p* < 0.001) (Table 4, Figure 2 and Figure 3). This suggests that there was a significant change in the level of cytokines contributing to the hyperactivity of the inflammatory response (Table 4).

## 4. Discussion

In this study, we observed that the changes in the reduction of immunological inflammation over a short 2 h exposure to the forest environment were very significant. According to previous studies, a therapeutic effect on blood pressure in the elderly was observed upon forest bathing for a duration of seven days [36], and two days of forest bathing reduced pro-inflammatory cytokines [31]. This suggests that individuals who lead busy lifestyles can reduce the effects of conditions or diseases associated with inflammation by exposure to a forest environment. A study compared rodents chronically exposed to clean air or polluted air and confirmed that inflammatory mediator gene expression was increased [27]. These genes included IL-1, IL-6, and TNF-α. Stress is related to the development of both depression and anxiety. Stress aggravates the development of clinical depression, and a number of preclinical studies have suggested that microglia play a role in depression and stress. In a previous study, morphological activation of residential microglia was induced by exposure to acute stress [37]. Inflammation in the nervous system, termed “neuroinflammation” has been observed in patients with psychiatric disorders [38]. Patients with depression show higher serum levels of pro-inflammatory cytokines than a normal group, including IL-1, IL-6, IL-8, IL-12, interferon-γ, and TNF-α [39,40]. The pro-inflammatory profile and magnified microglial activation were associated with the development of stress-induced anhedonia in susceptible mice [41]. An IL-18 (a pro-inflammatory cytokine) knockout mouse showed minimized stress-induced morphological microglial activation, indicating a role for IL-18 in relieving the stress response [37]. Animal models of stress are used to investigate the neurobiology of depression and anxiety. The neuronal activities of pro-inflammatory cytokines are mainly mediated by microglia. Previous reports have consistently shown that increased levels of pro-inflammatory cytokines and microglial activation are associated with schizophrenia [42,43,44]. Microglia primarily modulate innate immunity and are linked with most inflammatory processes in the central nervous system [45]. Under normal conditions, microglia are down-regulated within the equilibrium of inhibitory and stimulatory signals [46]. Activated microglia, which show up-regulated expression of various receptors, produce larger amounts of pro-inflammatory cytokines [47]. Since patients with inflammatory and autoimmune disorders, such as diabetes and fibromyalgia, present with depressive symptoms, depression may be related to inflammation [48,49,50,51,52,53]. In addition, short-duration exposure to the forest environment had an antioxidant effect based on the significantly increased GPx levels in the peripheral blood. Based on the these results, periodic forest visits, even for short periods, can reduce inflammation, psychiatric disorders due to neuronal damage, and depression due to chronic inflammatory diseases. There were two important limitations to this study. First, the 2 h duration of the wash-out period may not have been a sufficient period. Second, during an indoor experiment using phytoncide aroma therapy, independent variables were based on the olfactory stimulation of phytoncide. However, in this study, the forest environment was the independent variable and it was difficult to realistically control multiple sensory stimuli such as visual, auditory, and tactile stimuli in addition to the phytoncide effect.

## 5. Conclusions

These results suggest that after exposure to a forest environment, significant changes occurred in cytokine levels to reduce the hyperactivity of immune cells. Moreover, we observed a significant increase in the antioxidant effect.

## Figures and Tables

**Table 1:** Demographic characteristics of the experimental groups.

Parameter		Mean (Standard Deviation)		*p*
		Group A	Group B	
Total sample number		19	22	
Sex	Male	7	7	0.735
	Female	12	15	
Age (years)		22.37(1.54)	23.09(3.28)	0.384
Height (cm)		167.88(9.17)	165.67(7.42)	0.399
Weight (kg)		67.74(15.72)	60.51(11.85)	0.102
Smoking	Yes/No	3/16	5/17	0.576
Drinking	Yes/No	11/8	21/1	0.004 **
Student loan	Yes/No	6/13	5/16	0.583
Part-time job	Yes/No	11/8	10/11	0.516
Economic Status	Good	3	4	0.760
	Fair	14	17	
	Poor	2	1	

Note: ** *p* < 0.05.

**Table 2:** Experimental protocol for subjects exposed to the forest or urban environment.

Schedule	Group A		Group B	
	Item	Location	Item	Location
09:30–10:30	Description of study procedure and subject allocation (Seoul Paik Hospital)			
10:30–12:30	Pre-test	Seoul Paik Hospital (urban environment)	Pre-test	Pine Fragrant Forest (forest environment)
12:30–14:30	Wash-out period	Hwado-eup (rural environment)	Wash-out period	Hwado-eup (rural environment)
14:30–15:30	Transportation		Transportation	
15:30–17:30	Post-test	Gapyeong Pine Fragrant Forest (forest environment)	Post-test	Seoul Paik Hospital (urban environment)

**Table 3:** Effect of exposure to forest environment on SRI-MF and its sub-scales.

Variable	Subscales	Mean (Standard Deviation)		*t*	*p*
		Urban	Forest		
SRI-MF	Somatic symptoms	7.61 (6.02)	5.02 (5.16)	2.347	0.024 *
	Depressive symptoms	8.76 (5.73)	6.02 (5.18)	2.339	0.024 *
	Anger symptoms	5.34 (4.57)	4 (4.15)	1.630	0.111
	Total	23.68 (15.8)	16.56 (13.38)	2.351	0.024 *

Note: SRI-MF: stress response inventory-modified form, * *p* < 0.05.

**Table 4:** Effect of exposure to forest environment on serum pro-inflammatory cytokine and GPx levels.

Variable	Mean (Standard Deviation)		*t*	*p*
	Urban	Forest		
GPx ^❈^	2.21 (0.16) µM	2.26 (0.10) pg/mL	−2.039	0.048 *
IL-6 ^❈^	−0.18 (0.25) pg/mL	−0.15 (0.23) pg/mL	−1.089	0.283
IL-8 ^❈^	10.76 (2.87) pg/mL	9.21 (2.72) pg/mL	4.559	0.001 ***
TNF-α ^❈^	0.97 (0.17) pg/mL	0.87 (0.17) pg/mL	4.130	0.001 ***

Note: GPx: glutathione peroxidase; IL-6: interleukin-6; IL-8: interleukin-8; TNF- α: tumor necrosis factor alpha; ^❈^ Because of the skewness of the data, log-transformation of the variables was performed; * *p* < 0.05, *** *p* < 0.001.

## Abbreviations

The following abbreviations are used in this manuscript:

GPx	glutathione peroxidase
IFN	interferon
IL	interleukin
NK	Natural killer
SRI-MF	stress response inventory-modified form
TNF	tumor necrosis factor
